# Pathological features of lymph nodes around inferior mesenteric artery in rectal cancer: a retrospective study

**DOI:** 10.1186/s12957-021-02264-9

**Published:** 2021-05-18

**Authors:** Chunhui Jiang, Ye Liu, Chunjie Xu, Yanying Shen, Qing Xu, Lei Gu

**Affiliations:** 1 Department of Gastrointestinal Surgery, Renji Hospital, Shanghai Jiao Tong University School of Medicine, Shanghai, 200127 China; 2 Department of Pathology, Renji Hospital, Shanghai Jiao Tong University School of Medicine, Shanghai, 200127 China

**Keywords:** Rectal cancer, Lymph nodes metastasis, High ligation, Inferior mesenteric artery, Pathological features

## Abstract

**Objective:**

This study aimed to explore the pathological characteristics of lymph nodes around inferior mesenteric artery in rectal cancer and its risk factors and its impact on tumor staging.

**Methods:**

485 rectal cancer patients underwent proctectomy surgery were collected in this study. Clinical features of patients, including gender, age, BMI, tumor size, pathological type, differentiation, nerve invasion, lymph nodes, tumor marker, and pathological examinations, were analyzed.

**Results:**

A total of 485 cases were included in this study. There were 29 cases with IMA-LN metastasis; the metastasis rate was 5.98% (29/485). Positive IMA-LNs were associated with distance from anal verge, CEA, pathological type, differentiation, nerve invasion, T stage, and N stage. Multivariate analysis showed that distance from anal verge, CEA level, differentiation, and T stage were independent risk factors for positive IMA-LNs.

**Conclusion:**

Distance from anal verge, CEA level, differentiation, and T stage were independent risk factors for positive IMA-LNs. No skip metastasis occurred in IMA-LNs. We should choose the appropriate surgical methods to achieve better oncological results and reduce the incidence of postoperative complications.

## Introduction

In the treatment principle of colorectal cancer, there are great differences between the East and the West. For locally advanced rectal tumors, the US guidelines recommend TME surgery on sequence of neoadjuvant chemotherapy, while for resectable colon tumors, neoadjuvant chemotherapy plus intestinal resection and full dissection of regional lymph nodes are recommended. However, Asian doctors represented by China, Japan, and South Korea advocated D3 lymphadenectomy based on Japanese guidelines. Therefore, whether to perform the third station lymph node dissection is controversial. As the core of the third station lymph nodes, the one around the root of inferior mesenteric artery (IMA-LNs), its impact on prognosis and the way of dissection have been the focus of research.

## Materials and methods

### Clinical samples

From Jan. 2018 to Dec. 2020, we performed a retrospective analysis of patients who were eligible to receive proctectomy surgery in the Department of Gastrointestinal Surgery, Renji Hospital, Shanghai Jiao Tong University School of Medicine. Inclusive criteria: no distant metastasis, no obstruction, no emergency surgery, no radiotherapy or chemotherapy and other anti-tumor treatment, no history of other malignant tumors, and no colorectal multiple primary cancer. Routine MR examinations were performed before surgery, and clinical TN staging was performed. The study was approved by the Research Ethics Committee of Renji Hospital and carried out in accordance with the ethical standards formulated in the Helsinki Declaration.

Surgical procedure:

1. General anesthesia, adjust the patient’s position, and laparoscopic exploration.
2. Mobilize the sigmoid colon along the inferior mesenteric artery, find the Toldt’s fascia, and mobilize the lateral peritoneum.
3. Separate the root of the naked inferior mesenteric artery and clean the lymph nodes at the root of the inferior mesenteric artery.
4. Ligate the artery after being clamped by Hem-O-lock at the root of IMA and IMV about 2 cm, resected IMA-LNs.
5. Naked the intestine 10 cm proximal of the tumor and 5 cm at the distal of the tumor and transected, removed the tumor specimen, and anastomosed by circular stapler.

### Pathological analysis

The specimens were dissected by the surgeon after operation, and all the accessible lymph nodes were routinely submitted for examination. Pathological examination was performed by pathology department of our hospital. Routine examination included HE stained of tumor sections and lymph nodes and microsatellite instability (expression of mismatch repair protein MLH1, MSH2, MSH6, and PMS2) and ras gene mutation (K-ras, N-ras and BRAF gene mutations). Pathological staging was performed according to American Joint Committee on Cancer (AJCC) 8th Edition TNM staging system.

### Statistical analysis

All categorical data were counted as cases or percentages, and continuous data were expressed as mean ± SD. Statistical analyses were conducted by Statistical Product and Service Solutions (SPSS) 20.0 (Chicago, IL, USA) and GraphPad Prism 5 software. Categorical data were analyzed using the chi-squared (*χ*^2^) test or Fisher’s exact test. Multivariate analysis was performed through multivariate Cox proportional hazards regression analysis. Statistical significance was reached at a value of (P <0.05).

## Results

### Positive rate of IMA-LN

According to the above inclusion criteria, a total of 485 cases were enrolled in this study. All patients successfully completed IMA-LN dissection. In this study, IMA-LN metastasis (positive) was defined as at least one positive lymph node was found in this area. In IMA-LNs, a total of 1043 lymph nodes were found, of which 38 were positive (3.6%). The rate of IMA-LNs lymph node metastasis rate was defined as IMA-LN metastasis cases/total number of cases. Among all the included cases, there were 29 cases with IMA-LN metastasis; the metastasis rate was 5.98% (29/485).

### IMA-LN and clinical characters

In this study, we found that positive IMA-LNs were not associated with gender, age, or body mass index (BMI), but related to the distance from anal verge. All patients were routinely examined for tumor markers, including carcinoembryonic antigen (CEA), carbohydrate antigen 19-9 (CA19-9), and carbohydrate antigen 72-4 (CA72-4). We found that positive IMA-LNs were not associated with CA19-9 or CA72-4, but related to CEA (Table 1).

**Table 1:** IMA-LN and clinical characters

	Case (n)	IMA-LN		χ^2^ value	*P* value
		Metastasis (n)	Metastasis rate (%)		
Gender					
Male	296	16	5.4	0.445	0.505
Female	189	13	6.9		
Age (years)					
≤60	171	10	5.8	0.008	0.929
>60	314	19	6.1		
BMI (kg/m^2^)					
≤25	298	19	6.4	0.216	0.642
>25	187	10	5.3		
Distance from anal verge(cm)					
≤10	322	13	3.7	6.428	0.011*
>10	163	16	9.2		
CEA (ng/ml)					
≤5	202	5	2.5	7.561	0.006*
>5	283	24	8.5		
CA19-9 (U/ml)					
≤27	213	10	4.7	1.115	0.291
>27	272	19	7.0		
CA72-4 (U/ml)					
≤6.9	304	17	5.6	0.217	0.641
>6.9	181	12	6.6		

*P<0.05, the difference was statistically significant

### IMA-LN and pathological parameters

Positive IMA-LNs were not associated with tumor size, lymph nodes harvest number, microsatellite status, or ras phenotype, but related to pathological type, differentiation, and nerve invasion. And positive IMA-LNs were related to T stage and N stage, as shown in Table 2. Interestingly, there was no case of IMA-LN positive without paracancerous lymph node metastasis, which means no case of skip metastasis.

**Table 2:** IMA-LN and pathological parameters

	Case (n)	IMA-LN		χ^2^ value	*P* value
		Metastasis (n)	Metastasis rate (%)		
Tumor size(cm)					
≤4	262	11	4.2	3.215	0.073
>4	223	18	8.1		
Pathological type					
ADC	408	20	4.9	5.306	0.021*
MC/SRCC	77	9	11.7		
Differentiation					
Well/moderate	384	12	3.1	26.724	<0.0001*
Poor	101	17	15.9		
Nerve invasion					
No	381	11	2.9	30.22	<0.0001*
Yes	104	18	17.3		
LN harvest number					
<12	138	7	5.1	0.282	0.595
≥12	347	22	6.3		
Microsatellite status					
Stable	440	25	5.7	0.747	0.387
Unstable	45	4	8.9		
Ras phenotype					
Wild	289	17	5.8	0.012	0.918
Mutant	196	12	6.1		
T stage					
Tis or 1	16	0	0	35.72	<0.0001*
2	40	1	2.5		
3	286	12	4.2		
4^a^	143	16	11.2		
N stage					
0	206	0	0	10.43	0.015*
1	162	13	8.0		
2	117	16	13.7		

*ADC* adenocarcinoma, *MC* mucinous carcinoma, *SRCC* signet ring cell carcinoma

*P<0.05, the difference was statistically significant

^a^Including high rectal cancer and preoperative MRI prompt T3

Further, eight variables with P<0.1 (distance from anal verge, CEA level, tumor size, pathological type, differentiation, nerve invasion, T stage, and N stage) were included in the multivariate analysis. The analysis showed that distance from anal verge, CEA level, differentiation, and T stage were independent risk factors for positive IMA-LNs (Table 3).

**Table 3:** Multivariate logistic regression analysis for risk factors on IMA-LN metastasis

	Regression coefficients	Standard error	Wald value	OR	95% CI	*P* value
Distance from anal verge	−1.545	0.718	4.633	0.213	0.052–0.871	0.031*
CEA level	−2.721	0.980	7.710	0.066	0.010–0.049	0.005*
Tumor size	−0.481	0.777	0.383	0.618	0.135–2.835	0.536
Pathological type	0.003	0.806	0.000	1.003	0.207–4.869	0.997
Differentiation	−3.913	0.714	30.050	0.020	0.005–0.081	0.000*
Nerve invasion	−1.484	0.845	3.084	0.227	0.043–1.118	0.079
T stage	−2.169	1.078	3.883	0.121	0.015–1.045	0.048*
N stage	−0.672	0.586	1.316	1.958	0.621–6.167	0.251

*P<0.05, the difference was statistically significant

## Discussion

Lymph node metastasis is the most common and main metastasis pathway of colorectal cancer, and it is also an important indicator of staging and prognosis of colorectal cancer [1, 2]. The value of lymph node dissection around root of IMA is still disputed. Many studies have reported that D3 dissection can reduce paraaortic recurrence and systemic metastasis [3], and improve the prognosis [4, 5]. On the other side, some studies believe that the lymph node metastasis rate of IMA-LNs is relatively low, even after resection this kind of patients suggest poor prognosis, so it is of less significance to be resected [6–8].

Our study showed that the positive rate of IMA-LN was related to distance from anal verge, CEA level, tumor size, pathological type, differentiation, nerve invasion, T stage, and N stage. The result is similar to the previous reports [9]. Sun et al. [10] pointed out that for rectal cancer, neoadjuvant chemoradiotherapy can reduce the lymph node metastasis rate of IMA-LNs. For the patients who received neoadjuvant chemoradiotherapy, the location of the lesion above peritoneal reflexes, low degree of tumor differentiation and high preoperative serum CEA level were the risk factors of positive IMA-LNs. Nagasaki et al. [11] found that for patients with stage III colon cancer, serum CEA level, T stage, and number of lymph node dissection will significantly affect the positive situation of the third station lymph nodes (including IMA-LNs). Multivariate logistic regression analysis showed that only four factors (distance from anal verge, CEA level, differentiation, and T stage) were independent risk factors for positive IMA-LNs. There is no clear evidence that different locations of the lesion in the rectum affect the lymph node metastasis rate. It is worth noting that the lymph node metastasis rate of sigmoid colon tumor is significantly higher than that of rectal tumor [7, 12].

The AJCC staging is determined by the number of lymph nodes rather than the distance from the tumor [13, 14]. There are few studies and reports about the effect of IMA-LNs on TNM staging [15]. The positive IMA-LNs can aggravate the severity of the original stage III patients [16, 17]. Some surgeons believe that IMA-LNs metastasis can occur in T2, 3, 4 colorectal tumors, and there may be skip metastasis. Therefore, IMA-LNs should be routinely removed for colorectal tumors beyond T1 [18, 19]. But in this study, we did not find N positive was caused by only IMA-LN, which means no skip metastasis. It also means that if IMA-LNs turn negative, TNM staging will not be reduced. Does it mean that IMA-LN is not the origination of metastasis, but just the destination or interchange station?

Whether lymph node dissection around IMA can benefit patients is still uncertain, which may be the reason why the guidelines differ in this respect. Since there is no clear evidence that D3 lymph node dissection can benefit patients, the European and American guidelines do not consider it necessary to perform routine third station lymph node dissection [20]. High ligation has been reported to be effective in oncology; it can reduce paraaortic recurrence and systemic metastasis and improve the prognosis of some patients [21]. But from the point of view of complications such as anastomosis leakage and postoperative physiological dysfunction, it seems that high ligation is slightly worse than low ligation [22, 23]. For laparoscopic or robotic assisted radical surgery for colorectal cancer, the guidelines are conservative and not recommended as a routine recommendation. Only doctors with relevant experience should be recommended. At the same time, tumor staging, lymph node metastasis, and surgical difficulty should be considered comprehensively [24]. Many studies think that there is no significant difference between high and low ligation [25–27]. In our study, from the pathological features, the benefit of high ligation with low tumor location is limited.

Although the range of lymphadenectomy is controversial in different guidelines, the importance of lymphadenectomy is consistent. According to Japanese Society for Cancer of the Colon and Rectum (JSCCR) guidelines for the treatment of colorectal cancer, IMA-LNs are defined as the lymph nodes from the root of IMA to the beginning of LCA and along the IMA [28–30]. Similarly, follow the principles of CME, the scope of dissection is around the root of IMA, but it often goes beyond the boundary in real operation. It is possible that part of the retroperitoneal tissue may be removed due to excessive traction. So we need further research to define such a region.

Distance from anal verge, CEA level, differentiation, and T stage were independent risk factors for positive IMA-LNs. No skip metastasis occurred in IMA-LNs. Surgeons should fully evaluate the above-related factors and choose the appropriate surgical methods in order to achieve better oncological results and reduce the incidence of postoperative complications. At present, there is no strong evidence of evidence-based medicine that IMA-LNs dissection can improve the prognosis of patients, but IMA-LN metastasis is a risk factor for poor prognosis. With the development of precision medicine, it is expected that new diagnostic techniques can accurately evaluate the status of lymph node metastasis before operation, and more high-quality multicenter randomized controlled trial is expected to guide clinical decision-making.

As a retrospective analysis, some limitations existed in this study. First, in this study, the sample size is limited. If the sample size is increased, more accurate conclusions can be drawn. Second, a variety of statistical methods can be used to analyze the data and mutually verify the results.

## Data Availability

The datasets used and analyzed during the current study are available from the corresponding author on reasonable request.

## Abbreviations

IMA: Inferior mesenteric artery
IMV: Inferior mesenteric vein
LN: Lymph node
LCA: Left colonic artery
BMI: Body mass index
